# Suicidality and Its Associated Factors Among Students in Rural China During COVID-19 Pandemic: A Comparative Study of Left-Behind and Non-Left-Behind Children

**DOI:** 10.3389/fpsyt.2021.708305

**Published:** 2021-08-03

**Authors:** Tianya Hou, Xiaofei Mao, Xiaoqin Shao, Fen Liu, Wei Dong, Wenpeng Cai

**Affiliations:** ^1^Faculty of Psychology, Second Military Medical University, Shanghai, China; ^2^The Second Primary School, Shaoyang, China; ^3^Department of Psychology, Fudan University, Shanghai, China

**Keywords:** suicidal ideation, suicidal attempts, left-behind children, maladaptive strategies, depressive symptoms, anxious symptoms

## Abstract

**Introduction:** The coronavirus disease 2019 (COVID-19) has rapidly spread worldwide. The harmful impact of COVID-19 is beyond just physical health concern. The unprecedented public health crisis has also taken its toll on the mental health of adolescents. The present study aims to estimate the prevalence of suicidal ideation and attempts and investigate the similarities and differences in the influential factors for suicidal ideation and attempts among left-behind children (LBC) and non-left-behind children (NLBC) in rural China during the COVID-19 pandemic.

**Method:** A total of 761 rural Chinese students, of whom 468 were left behind, completed the cross-sectional questionnaires including demographic data, Cognitive Emotion Regulation Questionnaire, nine-item Patient Health Questionnaire, seven-item Generalized Anxiety Disorder Scale, suicidal ideation, and suicidal attempts. Chi-square test, independent-sample *t*-test, and logistic regression were performed in the statistical analysis.

**Results:** Overall, 36.4 and 10.4% of rural Chinese students reported suicidal ideation (37.8% for LBC vs. 34.1% for NLBC) and attempts (11.3% for LBC vs. 8.9% for NLBC) during the COVID-19 pandemic. Among LBC, parental educational level (adjusted odds ratio, Adj. OR = 1.60), maladaptive strategies (Adj. OR = 1.04), anxious symptoms (Adj. OR = 2.61), and depressive symptoms (Adj. OR = 3.85) were significantly associated with suicidal ideation, while age (Adj. OR = 0.56), maladaptive strategies (Adj. OR = 1.08), symptoms of anxiety (Adj. OR = 3.85), and symptoms of depression (Adj. OR = 2.68) were significantly related to suicidal attempts during the COVID-19 outbreak. Among NLBC, gender (Adj. OR = 2.20), parental educational level (Adj. OR = 1.77), perceived family economic status (Adj. OR = 0.39), anxious symptoms (Adj. OR = 2.38), and depressive symptoms (Adj. OR = 2.77) were significantly associated with suicidal ideation, whereas only anxious symptom (Adj. OR = 5.85) was significantly related to suicidal attempts.

**Conclusion:** Suicidal ideation and attempts are prevalent among students in rural China during the COVID-19 outbreak. Our findings also revealed the shared and unique factors for suicidal ideation and attempts among LBC and NLBC during the COVID-19 epidemic. With regard to the differences between LBC and NLBC, the use of maladaptive strategies and age might be vital factors for suicide prevention measures directed specifically toward LBC, whereas interventions sensitive to gender and perceived social economic status should be specifically designed for NLBC amid the COVID-19 pandemic.

## Introduction

Since December 2019, the coronavirus disease 2019 (COVID-19), caused by severe acute respiratory syndrome coronavirus 2 (SARS-COV-2), has rapidly spread worldwide and affected every continent (1). The harmful impact of COVID-19 is beyond just physical health concern (2). The unprecedented public health crisis has also taken its toll on the mental health of the public. Adolescence is a vulnerable stage, and adolescents experience a time of difficult transition, which makes them particularly susceptible to the detrimental effect of COVID-19 (3).

Suicide, as a major public health concern, is the second most leading cause of death among individuals aged 10–24 years (4, 5). Suicidal ideation and attempts are the most prominent precursors of complete suicide which is a preventable public health problem (6, 7). Suicidal ideation refers to the thoughts or impulses of committing suicide, while suicidal attempts refer to self-destructive behaviors with the intention to die (8). It has been reported that ~4 per 100,000 adolescents aged 10–19 die by suicide annually (9). Recent literature suggested that COVID-19 contributed to the higher risk of suicidality among adolescents (10). A priority agendum for the prevention of suicide in adolescents during COVID-19 is to identify the potential influential factors of suicidal ideation and attempts.

The influential factors for suicidal behaviors were multiple in origin, including psychological, biological, cognitive, social, and family factors (11, 12). A study examining the prevalence and factors of suicide among rural adolescents of China found that poor academic performance, life stress, external locus of control, symptoms of depression, and aggression were associated with the enhanced risk of suicide (11). Previous literature provided evidence for the association between negative emotional regulation strategies and suicidal behaviors. Difficulties with using cognitive reappraisal were found to be related to enhanced suicidal risk (13, 14). Additionally, anxious and depressive symptoms have been proven to be two independent risk factors for suicide (15, 16). Recent evidence suggested that quarantine experiences, enhanced psychological distress, unemployment, poor health-related information, and pre-existing sleep and mental problems were risk factors for suicidal ideation among the general population during the COVID-19 outbreak (17). Although many studies have presented the relationship between numerous psychosocial factors and suicidal behaviors as described above, the updated prevalence and influential factors for suicidal ideation and attempts among rural adolescents during the COVID-19 pandemic remain elusive.

A rural area refers to a geographic region that is located outside towns or cities with a low population density and small settlements (18). In a rural area, agriculture is the main source of livelihood, along with other primary industries. In recent years, China has experienced a rapid urbanization process, which contributes to a large-scale rural-to-urban migration. Rural residents migrate to cities to get employed on account of economic incentives (19). Over the recent decades, the number of rural-to-urban migrant workers has increased dramatically in China (20). The majority of rural migrant workers have to leave their children living in their hometown due to the high cost of living in urban cities and huge barriers to education and healthcare, resulting in the “left-behind children” phenomenon. Based on the available literature, left-behind children (LBC) are those aged 18 or below who continue to live in their hometown when one or both parents migrate to cities for work for at least 6 months (21, 22). According to a national survey conducted by the China Women's Federation in 2013, more than 60 million children in rural China are left behind, accounting for more than one-third of rural Chinese children and more than one-fifth of the entire population of children in China. The national survey also revealed an uneven distribution of the left-behind group in China, with LBC mainly gathering in mid-west China such as Sichuan Province and Anhui Province (14, 23). The findings regarding whether the rate of mental health problems is higher in LBC than in non-left-behind children (NLBC) are not homogenous in the previous literature. Some previous literature has suggested that, compared with NLBC, LBC presented a higher prevalence of psychological problems due to impaired parent–child bonding, reduced parental support, and weakened parental guidance (6, 24). However, some recent research also presented no significant difference in psychological status between LBC and NLBC (25–27).

In recent decades, there is growing empirical literature that investigated the mental health status and its associated factors among LBC; however, less is known regarding the difference in the updated prevalence of suicidal ideation and attempts and the influential factors for suicidal ideation and attempts among LBC and NLBC during the COVID-19 outbreak. The study aims to assess the prevalence of suicidal ideation and attempts among LBC and NLBC amid the COVID-19 pandemic and investigate the shared and unique factors influencing the suicidal ideation and attempts among LBC and NLBC.

## Method

### Participants

The cross-sectional study was conducted from August 1 to 5, 2020 in the rural areas of Anhui Province, which is a relatively under-developed and labor-exporting region (28). We randomly selected 15 classes from five senior high schools using two-stage random cluster sampling. In the first stage of sampling, five schools were randomly selected using a random number table. At the second stage of sampling, five classes were randomly selected from each school using a random number table. The inclusion criteria for the participants were as follows: (1) aged below 18 years old and (2) being born and raised in the countryside.

The participants were recruited through in-class invitation. Five well-trained investigators explained the purpose and procedure of the survey before starting it. The paper-based questionnaires were administrated in schools during classroom time. There is no incentive for completing the survey. A total of 780 students were invited to participate, with nine students refusing to answer the survey and 10 students returning incomplete survey. Finally, 761 participants were included in the analysis, including 468 LBC (61.5%) and 293 NLBC (38.5%).

The research processes were approved by the Research Ethics Commission of the Second Military Medical University and the permission to investigate was obtained from the local Education Bureau and school administrators. All the participants and their caregivers signed the informed consent form.

### Measurements

#### Socio-Demographic Variables

The sociodemographic characteristics obtained from the participants included age, gender, parental educational level, perceived family economic status, and left-behind or non-left-behind. With regard to parental educational level, we ranked it as low and high corresponding to elementary or below, high school or above, respectively. The LBC were considered to be participants with one or both parents having migrated to work for at least 6 months.

#### Maladaptive Emotion Regulation Strategies

Cognitive Emotion Regulation Questionnaire (CERQ) was used to measure emotion regulation strategies used to regulate emotion in response to stressful life events (29). The 36-item questionnaire consists of nine four-item subscales assessing different emotion regulation strategies, including four maladaptive strategies (self-blame, other-blame, rumination, and catastrophizing) and five adaptive strategies (positive focusing, planning, positive reappraisal, putting into perspective, and acceptance) (30). The participants were asked to rate how often they engage in each strategy on a five-point Likert scale, ranging from 1 “almost never” to 5 “almost always.” The scores of maladaptive (CERQ_M) strategies were calculated by summing the relevant subscales. The scale has been widely used in research with adolescents and showed good reliability and validity (31). In the present study, the Cronbach's alpha of CREQ_M was 0.852.

#### Anxious Symptoms

The Chinese version of the seven-item Generalized Anxiety Disorder Scale (GAD-7) was employed to measure symptoms of anxiety (32). The individuals were required to rate the frequency of each symptom during the past 2 weeks on a four-point scale ranging from 0 (not at all) to 3 (nearly every day). The sum scores of GAD-7 range from 0 to 21, with higher scores denoting more severe symptoms of anxiety. A cutoff of GAD-7 ≥10 was used to screen symptoms of anxiety (33). The scale has demonstrated good psychometric properties in adolescent samples (34, 35). In the present study, the Cronbach's alpha of GAD-7 was 0.913.

#### Depressive Symptoms

The Chinese version of the Patient Health Questionnaire-9 (PHQ-9) was utilized to measure depressive symptoms during the past 2 weeks. Each item was scored from 0 (not at all) to 3 (nearly every day), yielding a total score ranging from 0 to 27. A higher total score represented more severe symptoms of depression. The optimal dichotomization cutoff point on PHQ-9 was 10 (36). The scale has been widely used in measuring symptoms of depression in adolescents and presented adequate psychometric properties (35, 37). The Cronbach's alpha of PHQ-9 in the current study was 0.882.

#### Suicidal Ideation and Suicidal Attempts

Suicidal ideation and suicidal attempts were assessed with two items: “I thought about killing myself” and “I deliberately tried to kill myself,” which were derived from the Youth Self-Report questionnaire (38). The response options were “never,” “sometimes,” and “often” during the past month. The participants who chose “sometimes” or “often” on the first item were characterized as having suicidal ideation, and those who answered “sometimes” or “often” on the second item were considered to have suicidal attempts. This measure of suicidal ideation and suicidal attempts has been broadly used in prior research on adolescent suicidality (39, 40).

### Statistical Analysis

Firstly, descriptive analyses were performed to describe the demographic characteristics of the respondents. The differences in the variables including demographic variables among LBC and NLBC were assessed by chi-square test and independent-sample *t*-test. Secondly, univariate logistic regression was employed to evaluate the univariate associations of demographic variables, maladaptive strategies, anxious symptoms, and depressive symptoms with suicidal ideation and attempts among LBC and NLBC, respectively. Finally, multivariate logistic regression models were conducted to investigate the potential influential factors for suicidal ideation and suicidal attempts among LBC and NLBC, respectively. Adjusted odds ratios (OR) and 95% confidence interval (95% CI) were reported. Statistical analysis was performed using Statistical Package for Social Sciences (SPSS), version 25.0. All variables were binary, and statistical significance was defined as *P* < 0.05 (two-sided tests).

## Results

### Demographic Characteristics

The sample characteristics are shown in Table 1. The age of the participants ranged from 14 to 18 years old, with a mean age of 16.09 ± 0.61 years old. Approximately 15% of the sample perceived the financial status of their family to be below average. A considerable proportion of the sample had symptoms of anxiety (24.0%) and depression (27.7%) during the COVID-19 outbreak. The self-reported 6-month prevalence of suicidal ideation and attempts among adolescents in rural China was 36.4 and 10.4% during the COVID-19 pandemic. No significant differences in age, gender, parental educational level, scores of maladaptive strategies, anxious symptoms, depressive symptoms, suicidal ideation, and suicidal attempts between LBC and NLBC were found.

**Table 1:** The demographic characteristics of the left-behind children (LBC) and non-left-behind children (NLBC) groups; mean ± SD or no. (%).

**Variables**	**Total** ** (*n* = 761)**	**LBC** ** (*n* = 468)**	**NLBC** ** (*n* = 293)**	***X*^**2**^/*t***
Total	761 (100)	468 (61.5)	293 (38.5)	
Age (years)	16.09 (0.61)	16.10 (0.60)	16.07 (0.64)	0.799
Gender				0.50
Male	451 (59.3)	282 (60.3)	169 (57.7)	
Female	310 (40.7)	186 (39.7)	124 (42.3)	
Parental educational level				0.28
Low	378 (49.7)	236 (50.4)	142 (48.5)	
High	383 (50.3)	232 (49.6)	151 (51.5)	
Perceived family economic status				0.37
Below average	112 (14.7)	66 (14.1)	46 (15.7)	
Average/above average	649 (85.3)	402 (85.9)	247 (84.3)	
Scores of maladaptive strategies	41.37 (10.29)	41.64 (10.22)	40.94 (10.42)	0.91
Anxiety				1.27
No	578 (76.0)	349 (74.6)	229 (78.2)	
Yes	183 (24.0)	119 (25.4)	64 (21.8)	
Depression				0.76
No	550 (72.3)	333 (71.2)	217 (74.1)	
Yes	211 (27.7)	135 (28.8)	76 (25.9)	
Suicidal ideation				1.06
No	484 (63.6)	291 (62.2)	193 (65.9)	
Yes	277 (36.4)	177 (37.8)	100 (34.1)	
Suicidal attempts				1.16
No	682 (89.6)	415 (88.7)	267 (91.1)	
Yes	79 (10.4)	53 (11.3)	26 (8.9)	

### Factors Influencing the Suicidality of Rural Adolescents During the COVID-19 Pandemic

#### Univariate Analysis

The univariate associations of predictors with suicidal ideation and attempts are presented in Tables 2, 3. Females were more likely to report suicidal ideation than males in both LBC and NLBC groups (OR = 1.55, 95% CI: 1.06–2.27 and OR = 2.06, 95% CI: 1.26–3.37). Across the overall sample, participants with a high parental educational level had a significantly higher likelihood of suicidal ideation (OR = 1.43, 95% CI: 1.06–1.92). Nevertheless, this difference was not significant in the LBC and NLBC groups. In the overall sample, adolescents who rated their financial status as average or above average were at a lower risk of having suicidal ideation than those who rated their financial status as below average (OR = 0.59, 95% CI: 0.39–0.88). However, this discrepancy disappeared in the LBC group. For both LBC and NLBC groups, high levels of maladaptive strategies were associated with a higher likelihood of suicidal ideation (OR = 1.07, 95% CI: 1.05–1.09 and OR = 1.05, 95% CI: 1.02–1.07). Anxious and depressive symptoms were positively associated with suicidal ideation not only in the LBC group (OR = 6.61, 95% CI: 4.17–10.47 and OR = 8.07, 95% CI: 5.14–12.68) but also in the NLBC group (OR = 4.69, 95% CI: 2.61–8.43 and OR = 4.28, 95% CI: 2.47–7.41).

**Table 2:** Univariate logistic regression results of the association between suicidal ideation and predictors.

	**Total**	**Non-left-behind** ** children**	**Non-left-behind** ** children**
Age (years)	0.97 (0.76, 1.23)	0.99 (0.72, 1.35)	0.94 (0.64, 1.38)
Gender			
Male	1.00	1.00	1.00
Female	1.72 (1.27, 2.32)	1.55 (1.06, 2.27)	2.06 (1.26, 3.37)
Parental educational level			
Low	1.00	1.00	1.00
High	1.43 (1.06, 1.92)	1.40 (0.96, 2.04)	1.49 (0.91, 2.42)
Perceived family economic status			
Below average	1.00	1.00	1.00
Average/above average	0.59 (0.39, 0.88)	0.69 (0.41, 1.17)	0.45 (0.24, 0.86)
Scores of maladaptive strategies	1.06 (1.04, 1.08)	1.07 (1.05, 1.09)	1.05 (1.02, 1.07)
Anxiety			
No	1.00	1.00	1.00
Yes	5.84 (4.07, 8.38)	6.61 (4.17, 10.47)	4.69 (2.61, 8.43)
Depression			
No	1.00	1.00	1.00
Yes	6.33 (4.47, 8.95)	8.07 (5.14, 12.68)	4.28 (2.47, 7.41)

**Table 3:** Univariate logistic regression results of the association between suicidal attempts and predictors.

	**Total**	**Left-behind** ** children**	**Non-left-behind** ** children**
Age (years)	0.80 (0.54, 1.16)	0.76 (0.47, 1.23)	0.83 (0.43, 1.48)
Gender			
Male	1.00	1.00	1.00
Female	1.75 (1.10, 2.80)	1.82 (1.03, 3.24)	1.67 (0.74, 3.74)
Parental educational level			
Low	1.00	1.00	1.00
High	1.60 (1.00, 2.58)	1.38 (0.77, 2.45)	2.27 (0.95, 5.39)
Perceived family economic status			
Below average	1.00	1.00	1.00
Average/above average	0.59 (0.33, 1.05)	0.67 (0.32, 1.41)	0.46 (0.18, 1.18)
Scores of maladaptive strategies	1.10 (1.07, 1.12)	1.11 (1.08, 1.15)	1.07 (1.03, 1.11)
Anxiety			
No	1.00	1.00	1.00
Yes	9.92 (5.92, 16.63)	9.39 (4.98, 17.71)	10.81 (4.43, 26.36)
Depression			
No	1.00	1.00	1.00
Yes	7.73 (4.63, 12.89)	9.26 (4.82, 17.77)	5.52 (2.38, 12.80)

Across the total sample, females were more likely to attempt suicide (OR = 1.75, 95% CI: 1.10–2.80). However, these differences were not significant in the NLBC group. For both LBC and NLBC groups, students with high scores of maladaptive strategies presented higher rates of suicidal attempts (OR = 1.11, 95% CI: 1.08–1.15 and OR = 1.07, 95% CI: 1.03–1.11). In the overall sample, symptoms of anxiety and depression were positively related to suicidal attempts (OR = 9.92, 95% CI: 5.92–16.63 and OR = 7.73, 95% CI: 4.63–12.89).

#### Multivariate Analysis

The results of multivariate logistic regression analyses are presented in Tables 4, 5. Three models were conducted to explore the influential factors influencing suicidal ideation and attempts among the total sample, LBC, and NLBC, respectively. After adjusting for demographic characteristics, symptoms of anxiety and depression were associated with a higher risk of suicidal ideation in both LBC (OR = 2.61, 95% CI: 1.474.62 and OR = 3.85, 95% CI: 2.236.67) and NLBC groups (OR = 2.38, 95% CI: 1.12–5.06 and OR = 2.77, 95% CI: 1.40–5.48). For both LBC and NLBC groups, students with a high parental educational level were more likely to report suicidal ideation (OR = 1.60, 95% CI: 1.02–2.49 and OR = 1.77, 95% CI: 1.01–3.12). A high score of maladaptive strategies remained negatively related to suicidal ideation in multivariate analysis among LBC only (OR = 1.04, 95% CI: 1.02–1.07). For NLBC only, the odds ratios of reporting suicidal ideation likewise remained higher among females than males (OR = 2.20, 95% CI: 1.27–3.79) and lower among adolescents with a perception of average/above average financial status than those with a perception of below average financial status (OR = 0.39, 95% CI: 0.19–0.82).

**Table 4:** Results of the multivariate logistic regression analyses predicting suicidal ideation among the total sample, left-behind children (LBC), and non-left-behind children (NLBC).

	**Model 1–Total**	**Model 2–LBC**	**Model 3–NLBC**
Age (years)	0.91 (0.69, 1.20)	0.83 (0.57, 1.21)	0.97 (0.64, 1.48)
Gender			
Male	1.00	1.00	1.00
Female	1.72 (1.23, 2.41)	1.44 (0.92, 2.23)	2.20 (1.27, 3.79)
Parental educational level			
Low	1.00	1.00	1.00
High	1.61 (1.14, 2.26)	1.60 (1.02, 2.49)	1.77 (1.01, 3.12)
Perceived family economic status			
Below average	1.00	1.00	1.00
Average/above average	0.62 (0.39, 0.99)	0.91 (0.48, 1.73)	0.39 (0.19, 0.82)
Scores of maladaptive strategies	1.03 (1.01, 1.05)	1.04 (1.02, 1.07)	1.01 (0.99, 1.04)
Anxiety			
No	1.00	1.00	1.00
Yes	2.42 (1.55, 3.79)	2.61 (1.47, 4.62)	2.38 (1.12, 5.06)
Depression			
No	1.00	1.00	1.00
Yes	3.39 (2.22, 5.16)	3.85 (2.23, 6.67)	2.77 (1.40, 5.48)

**Table 5:** Results of the multivariate logistic regression analyses predicting suicidal attempts among the total sample, left-behind children (LBC), and non-left-behind children (NLBC).

	**Total**	**LBC**	**NLBC**
Age (years)	0.69 (0.45, 1.05)	0.56 (0.32, 0.97)	0.87 (0.43, 1.75)
Gender			
Male	1.00	1.00	1.00
Female	1.82 (1.07, 3.11)	1.79 (0.91, 3.54)	1.74 (0.71, 4.29)
Parental educational level			
Low	1.00	1.00	1.00
High	1.65 (0.98, 2.87)	1.50 (0.76, 2.95)	2.33 (0.87, 6.28)
Perceived family economic status			
Below average	1.00	1.00	1.00
Average/above average	0.78 (0.39, 1.55)	1.17 (0.46, 2.95)	0.42 (0.14, 1.28)
Scores of maladaptive strategies	1.05 (1.03, 1.08)	1.08 (1.04, 1.21)	1.02 (0.97, 1.06)
Anxiety			
No	1.00	1.00	1.00
Yes	4.15 (2.17, 7.95)	3.85 (1.73, 8.59)	5.85 (1.86, 18.33)
Depression			
No	1.00	1.00	1.00
Yes	2.44 (1.26, 4.71)	2.68 (1.16, 6.19)	1.99 (0.66,6.00)

After controlling for confounders, symptoms of anxiety were strongly associated with the risk of suicidal attempts in both LBC and NLBC groups (OR = 3.85, 95% CI: 1.73–8.59 and OR = 5.85, 95% CI: 1.86–18.33). Age was a significant predictor of suicidal attempts in the LBC group only (OR = 0.56, 95% CI: 0.32–0.97). Overall, females were nearly twice as likely to report suicidal attempts as males (OR = 1.82, 95% CI: 1.07–3.11). Nonetheless, this was not found in either group. Higher scores of maladaptive strategies and symptoms of depression were positively associated with a greater risk of suicidal attempts within the overall sample (OR = 1.05, 95% CI: 1.03–1.08 and OR = 2.44, 95% CI: 1.26–4.71). However, the discrepancy was not significant in the NLBC groups.

## Discussion

This cross-sectional study revealed that the rates of suicidal ideation and attempts were 36.4 and 10.4% in a sample of rural Chinese students during the COVID-19 outbreak. In a recent study conducted 3 months earlier than our study, the rates of suicidal ideation and attempts among senior high school students in rural China were 31.3 and 7.5% (41), which is lower than our findings. A more recent meta-analysis presented the prevalence of suicidal ideation and attempts as 14.5 and 12.7% in individuals aged 12–15 years across 46 low- and middle-income countries (42), which is far less than the estimates reported in the present study. This indicates that the rates of suicidal ideation and attempts among rural Chinese children were extremely alarming during the COVID-19 pandemic and should be taken very seriously as a public health priority. The rates of anxious symptoms, depressive symptoms, and suicidal ideation and attempts were similar in both LBC and NLBC groups during the outbreak of COVID-19. Although this is consistent with recent findings that suggested that the damaging impact of left-behind was limited to the physical aspect of health and no significant difference in suicidality between LBC and NLBC was observed (26, 27), our results deserve further discussion. Our findings indicate that not only LBC but also NLBC are vulnerable to suicidality during the COVID-19 epidemic. The majority of the existing literature have focused on the mental health problems of LBC, while the mental health of NLBC has been relatively neglected in the rural mental health literature. Thus, our findings indicate that attention should not only be paid to the mental health problems of LBC but also be paid to the psychological status of NLBC. The present study also provided robust evidence suggesting that there was no significant difference in the characteristics between LBC and NLBC. Notably, our results presented that, in both LBC and NLBC groups, the majority perceived their family's financial status to be average or above average. Parents of NLBC are usually content with the income they earned and choose to stay in rural areas. Migrant workers get better employment opportunities with higher income, and LBC could receive relatively more remittances from their migrant parents (26, 43). Thus, both LBC and NLBC would perceive better financial status.

More than describing the current situation of suicidal ideation and attempts in rural Chinese children, our findings also revealed the potential risk and protective factors of suicidality in LBC and NLBC during the COVID-19 pandemic, respectively. For both LBC and NLBC, anxious symptoms were positively associated with suicidal ideation and attempts, which is consistent with the previous literature (15, 44). In our study, symptoms of depression predicted suicidal ideation in both LBC and NLBC groups, while depressive symptoms were only associated with suicidal attempts in LBC. This result is intriguing and warrants further investigation and replication. Both LBC and NLBC with a better educated parent were more likely to report suicidal ideation. This echoes the existing evidence during COVID-19 (45). Better educated parents remained busy with their jobs even during the pandemic and had less time to communicate with their children, which might increase the risk of experiencing mental health problems. Additionally, among LBC only, maladaptive strategy was a risk factor for suicidal ideation, and age and maladaptive strategies were influential factors for suicidal attempts, indicating the unique stress faced by LBC. Older LBC predicted a lower likelihood of suicidal attempts in our study, which contradicts the previous findings (42). The disparity might be attributed to the different age range of the participants. For example, a recent study reported that older age was associated with higher odds of suicidal behaviors. However, the age span of the participants was only from 12 to 15, and the study lacked the data on rural children aged 16–18 (42). The use of maladaptive strategies was only associated with suicidality among LBC. Compared with LBC, NLBC might have experienced more parental supervision (6), which could reduce the negative effect of maladaptive emotional regulation strategies on suicidality. Furthermore, gender and perceived family economic status were related to suicidal ideation only in the NLBC group. Consistent with the previous literature (6, 46), female NLBC were more likely to report suicidal ideation than their male counterparts. Previous evidence suggested that girls tended to be more sensitive to interpersonal relationships, and distinct hormone changes in girls vs. boys during pubertal maturation might also account for the disparity (6, 47). A perceived higher family economic status was associated with decreased odds of suicidal ideation, which is in line with previous literature suggesting the protective role of socioeconomic status in mental health (48).

Several limitations should be mentioned. Firstly, the present study employed a cross-sectional design, which cannot be used to make causal inferences. Future researchers might conduct a longitudinal research to explore the mechanisms of how influential factors result in suicidality among rural children in China. Secondly, the research involved self-reported questionnaires, and response bias might undermine the accuracy of the findings. Future study might collect information from diverse informants (e.g., teachers or parents). Thirdly, in the present study, we did not distinguish between rural children with one parent migrating and rural children with two parents migrating, and this therefore needs to be considered in further studies. Fourthly, all the subjects were recruited from schools, which might result in selection bias since LBC might have dropped out of school before completing compulsory education. Finally, the participants in our study were only from a rural area of Anhui province, which might restrict the generalization of the results to children in other rural areas of China.

To the best of our knowledge, our study is the first to provide an updated insight into the prevalence and the influential factors of suicidal ideation and attempts in LBC and NLBC during the COVID-19 pandemic. The rates of suicidal ideation and attempts were extremely high in both LBC and NLBC groups amid the pandemic. The study also highlighted the differences in risk factors for suicidal ideation and attempts between LBC and NLBC, which could help design targeted interventions to prevent suicidality among rural Chinese students.

## Data Availability Statement

The raw data supporting the conclusions of this article will be made available by the authors, without undue reservation.

## Ethics Statement

The studies involving human participants were reviewed and approved by Second Military Medical University. Written informed consent to participate in this study was provided by the participants' legal guardian/next of kin.

## Author Contributions

TH contributed to the writing of this article and the statistical analysis and is the first author. WC and WD led the whole study, including carrying out this study, and putting forward the study. XM, XS, and FL contributed to the data collection and statistical analysis. All authors contribute to the editing of the manuscript and have approved the final manuscript.

## Conflict of Interest

The authors declare that the research was conducted in the absence of any commercial or financial relationships that could be construed as a potential conflict of interest.

## Publisher's Note

All claims expressed in this article are solely those of the authors and do not necessarily represent those of their affiliated organizations, or those of the publisher, the editors and the reviewers. Any product that may be evaluated in this article, or claim that may be made by its manufacturer, is not guaranteed or endorsed by the publisher.
